# Remote tactile sensing system integrated with magnetic synapse

**DOI:** 10.1038/s41598-017-17277-2

**Published:** 2017-12-05

**Authors:** Sunjong Oh, Youngdo Jung, Seonggi Kim, SungJoon Kim, Xinghao Hu, Hyuneui Lim, CheolGi Kim

**Affiliations:** 1 Department of Nature-Inspired Nanoconvergence Systems, Korea Institute of Machinery and Materials, Daejeon, 34103 Republic of Korea; 2 Department of Emerging Materials Science & Center for Bio-Convergence Spin System, DGIST, Daegu, 42988 Republic of Korea

## Abstract

Mechanoreceptors in a fingertip convert external tactile stimulations into electrical signals, which are transmitted by the nervous system through synaptic transmitters and then perceived by the brain with high accuracy and reliability. Inspired by the human synapse system, this paper reports a robust tactile sensing system consisting of a remote touch tip and a magnetic synapse. External pressure on the remote touch tip is transferred in the form of air pressure to the magnetic synapse, where its variation is converted into electrical signals. The developed system has high sensitivity and a wide dynamic range. The remote sensing system demonstrated tactile capabilities over wide pressure range with a minimum detectable pressure of 6 Pa. In addition, it could measure tactile stimulation up to 1,000 Hz without distortion and hysteresis, owing to the separation of the touching and sensing parts. The excellent performance of the system in terms of surface texture discrimination, heartbeat measurement from the human wrist, and satisfactory detection quality in water indicates that it has considerable potential for various mechanosensory applications in different environments.

## Introduction

Tactile sensation recognizing from surrounding environment through the direct contact of force, vibration and temperature, is regarded as a next-generation technology for applications in information gathering and transfer as well as artificial intelligence^1–4^. In particular, biomimetic tactile sensors have attracted considerable attention as the core technology for replacing human sensing in minimally invasive surgery, health monitoring, robotics, virtual reality, and prosthetics^5–10^. Moreover, in the near future, various types of manufacturing robots are expected to co-operate with human workers in factories, and tactile sensing is a key technology for ensuring the safety of human workers and natural motion of robots^11^.

Various types of tactile sensors have been developed using Si-based micro-electro-mechanical system (MEMS) technologies. Recently, flexible tactile sensing systems have been demonstrated using elastomer composites and thin polymer film. Such flexible sensors can detect changes in contact resistance^12,13^, piezo-resistance^14,15^, piezo-electricity^16–18^, capacitance^10,19,20^, and triboelectricity^21,22^ by deformation of the sensing elements or electrodes under external stress. However, sensors based on flexible bulk elastomers suffer from high hysteresis, low sensitivity, limited sensing range, temperature-dependent displacement, and polymer swelling^23,24^ due to the intrinsic viscoelastic properties of elastomer composites^25^. To overcome these shortcomings, tactile sensors have been developed using microstructures or nanostructures, such as micropyramids^19^, interlocked microdomes^26^, and nanowires^15^. However, most tactile sensors are designed to achieve a trade-off between their sensitivity and their dynamic working range. Thus, highly sensitive tactile sensors have a limited dynamic range; conversely, sensors with a wide dynamic range have low sensitivity. Therefore, the development of universal tactile sensors with high sensitivity in wide working range is one of the major challenging issues for use in practical applications such as robotic skin, medical diagnostic devices, and bionic arm electronic skin (Table S1).

Another major issue is the development and application of flexible tactile sensors with robust interconnections^27^. Several interesting approaches have been proposed for attaching flexible electrodes to human skin or fabrics via silver nanowires^28^, liquid metals^29^, and sinuous thin Si ribbons^30^ for reliable performance under stretching and bending. In addition, a tactile pressure sensor based on an ultrathin substrate that is insensitive to mechanical bending and even crushing has been reported^31^. However, stable interconnection of wires still remains a challenge for developing tactile sensors with repeatability and robustness. Severe signal disturbances due to external electrical, magnetic, and thermal noise lead to various problems, as the external pressure contact sites are locates close to the physical sensing elements. Therefore, tactile sensors have limited applications in water or extreme environments unless they are provided with extensive packaging of the sensing elements.

Human skin is a fascinating tactile sensing system having multiple functions for detecting external stimulations, such as pressure, shear force, stretching, sliding, vibration, and temperature, with high accuracy and reliability. In human beings, tactile sensing is performed by the central nervous system when sensing signals from the mechanoreceptors inside the fingertips are transmitted through nerve cells by contactless methods in the synapses^26,32,33^. Inspired by human tactile sensing and synaptic transmission, this paper proposes a remote tactile sensing system integrated with a magnetic synapse to overcome the above-mentioned issues of flexible tactile sensors. The developed remote tactile sensing system consists of a remote touch tip that generates air pressure by external touch, an air tube that delivers the generated air pressure, and a magnetic synapse that transduces the air pressure into electrical signals. Specifically, the magnetic field intensity changes as the thin elastomer membrane is deflected by air pressure, which varies under external stress from the remote touch tip and is transmitted through the air tube. The remote touch tip, which has no electronic component, provides robustness and its tactile sensing capability is not affected significantly by external electrical, magnetic, and thermal noise even in water or harsh environments.

The magnetic synapse is composed of a thin elastomer membrane with an embedded permanent magnet above a magnetoresistive (MR) sensing element. It exhibits the most fascinating characteristics of remote tactile sensing from the viewpoints of both high sensitivity (30 mg) and a wide dynamic range (6 Pa ~400 kPa). Moreover, the sensitivity and dynamic range are controlled simply by modifying the structure of the thin magnetic layers in the MR sensing element. Thus, the developed remote tactile sensing system has high sensitivity and excellent durability over a wide dynamic pressure range without any hysteresis. Moreover, it can successfully detect surface texture differences, sense the heart rate from the human wrist, and is operable even in water.

**Figure 1:**
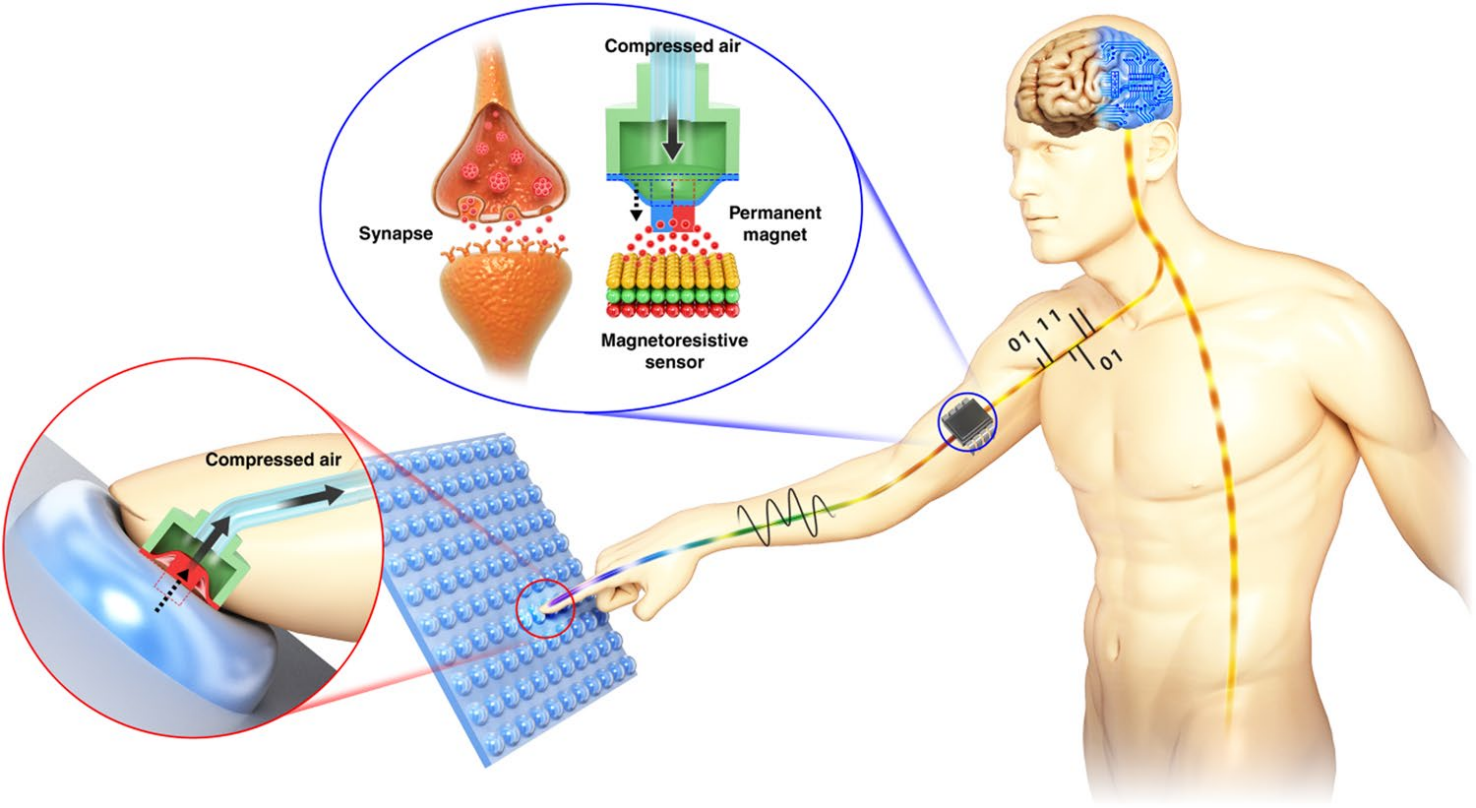
Schematic illustration of remote tactile sensing system with magnetic synapse inspired by human tactile sensing and synaptic transmitter. Mechanoreceptors in a fingertip recognize external tactile stimulations and convert them into electrical signals. The electrical signals are transmitted through the nervous system via synaptic transmitters and perceived by the brain. The developed remote tactile sensing system has a magnetic synapse that transmits tactile inputs in the form of air pressure variations, causing displacement of the position of the permanent magnet in the magnetic synapse. The displacements are perceived by a magnetoresistive (MR) sensing element and converted into electrical signals.

Figure 1 shows the working principle of the remote tactile sensing system inspired by human tactile sensing and synaptic transmission. When there is an external tactile stimulation, the mechanoreceptors in the human fingertip generate bioelectrical signals accordingly. These signals are transmitted by neural synapses to the central nervous system and recognized as tactile stimulations in the brain. In the remote tactile sensing system, an external tactile stimulation is transduced into air pressure variation in the remote touch tip. These air pressure variations are transmitted to the magnetic synapse through the air tube and transformed into electrical signals in the MR sensing element (Fig. S1). The magnetic synapse senses the tactile stimulation by measuring the variation of the magnetic field intensity. Physical separation of the remote touch tip and the magnetic synapse, as in the case of human tactile sensing, makes the remote tactile sensing system robust against external electromagnetic and thermal noise. Thus, it can be operated in water and other extreme environments. Furthermore, robust and hysteresis-free remote sensing is achieved by avoiding direct wiring in the touching part.

**Figure 2:**
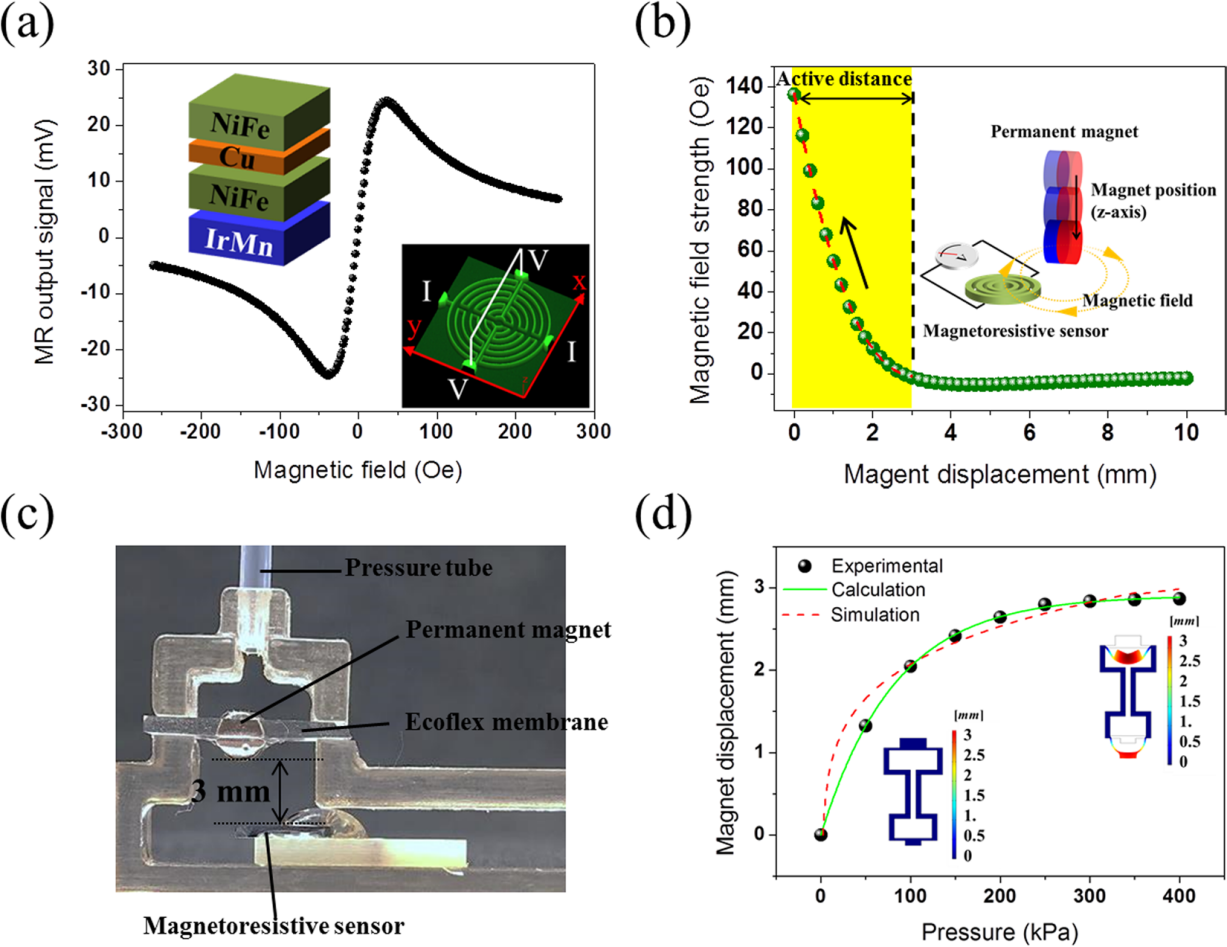
MR sensing element characteristics for magnetic synapse. (**a**) Output signal profile of MR sensing element measured by four-probe method. The external magnetic field was applied along the x-axis perpendicular to the exchange bias field direction. The MR sensing element consists of Ta/NiFe/Cu/NiFe/IrMn/Ta thin films with a spin-valve structure. The magnetic field sensitivity was 2.79 mV/Oe in the range of +300 Oe. The inset shows an optical 3D image of the multi-ring MR sensing element. (**b**) Finite element simulation (ANSYS, Maxwell 16.1) showing the correlation between the vertical position of the permanent magnet and the magnetic field strength present at the MR sensing element. The simulation was carried out to determine the optimum initial position of the permanent magnet relative to the MR sensing element. The magnetic field strength decreases as the vertical distance of the permanent magnet changes from 0 mm to 3 mm, which is categorized as the active distance. (**c**) Cross-sectional image of magnetic synapse with air tube connected. The initial position of the permanent magnet was 3 mm from the MR sensor, as determined by finite element simulation. (**d**) Permanent magnet displacement calculated from the measured output signal of the MR sensing element versus applied tactile pressure. Finite element simulation was carried out by COMSOL Multiphysics 5.2, hyperelastic module.

The controllable design of the magnetic layers in the MR sensing element provides the tactile sensing system with a wide dynamic working range and high sensitivity. The characteristics of the MR sensing element and magnetic synapse are explained in detail in Fig. 2. The remote touch tip has an air chamber capped by a polydimethylsiloxane (PDMS) membrane with a hemispherical tip at the center. Under external stimulation of the remote touch tip, the air in the chamber is compressed owing to deformation of the PDMS membrane. The compressed air is transferred through the air tube to the air chamber of the magnetic synapse. The magnetic synapse is composed of an MR sensing element and an air chamber capped by an Ecoflex^®^ 0030 membrane to enhance the pressure sensitivity (Fig. S2). The Ecoflex membrane has an embedded permanent magnet whose position changes as the Ecoflex membrane is deflected by air pressure variations generated in the remote touch tip by external stimulations. These movements induce variations in the magnetic field strength of the MR sensing element, which generates electrical output signals accordingly.

The overall sensitivity of the remote tactile sensing system is determined primarily by two major design considerations. The first major consideration is the design of the MR sensing element. The MR sensing element consists of multiple thin magnetically active layers that can control its sensitivity to external magnetic fields. Previously, we developed a highly sensitive hybrid MR sensing element, which combines anisotropic magnetoresistive (AMR) and planar Hall effects (PHE), consisting of multi-ring structures (diameter, 300 µm) in a hybrid Wheatstone bridge^34–36^. The MR sensing element was fabricated using DC magnetron sputtering and a lift-off process. After multi-ring patterning using a photolithography process, magnetic multilayers (spin-valve structure) of Ta(5 nm)/NiFe(3 nm)/Cu(1.2 nm)/NiFe(10 nm)/IrMn(10 nm)/Ta(5 nm) were deposited by a DC magnetron sputtering system. During the deposition, a uniform in-plane magnetic field of 250 Oe was applied to induce an exchange bias field at the interface between ferromagnetic NiFe and antiferromagnetic IrMn. The MR sensing element was characterized under external magnetic fields in the range of −300 to +300 Oe with a source current of 1 mA. The output voltage of the MR sensing element increases linearly between −35 Oe and +35 Oe with a high sensitivity of 1.15 mV/Oe (Fig. 2a). The magnetic field sensitivity of the MR sensing element is represented by a fitting function of the voltage and magnetic field. Thus, the sensitivity and dynamic range of the sensing element to external magnetic fields over a linear region can be controlled by modifying the magnetic multilayer structure (Figs S3 and S4). Therefore, the overall sensitivity and dynamic range of the remote tactile sensor are tailored by incorporating different types of MR sensing elements according to the external pressure level. Moreover, the characteristics of the MR sensing elements are reliable because they are manufactured using MEMS technologies. In addition, the fabrication procedure is conducive to mass production.

The second design consideration is the initial position of the permanent magnet with the magnetic synapse. Finite element simulation (ANSYS, Maxwell 16.1) was carried out to analyze the magnetic field strength produced by a cylindrical permanent magnet (ϕ3 mm × 2 mm). Figure 2b shows the magnetic field strength (H) present at the MR sensing element as the permanent magnet moves in a direction normal to the MR sensing element (more details in Fig. S5). It is obvious that the magnetic field strength decreases drastically from 140 to 0 Oe as the vertical distance (D) of the permanent magnet increases from 0 mm to 3 mm with the fitting function H = *g*_*n*_(*D*), and it remains nearly constant beyond 3 mm (see supplementary materials for function *g*_*n*_(*D*)). The permanent magnet embedded in the membrane moves closer to the MR sensing element as air pressure is applied to the membrane. To optimize the sensitivity and dynamic range of the remote tactile sensing system, the initial vertical distance of the permanent magnet relative to MR sensing element should be less than 3 mm, which is categorized as the active distance.

Figure 2c shows a cross-sectional image of the assembled magnetic synapse in the remote tactile sensing system. The thickness of the membrane of the magnetic synapse is 500 μm. The diameter and thickness of the cylindrical permanent magnet embedded in the membrane are 3 mm and 2 mm, respectively. The chamber is fabricated by a 3D printer and capped by the membrane. The initial position of the permanent magnet is 3 mm from the MR sensing element, as determined by finite element simulation (Fig. 2b). The calculated and simulated positions of the permanent magnet under varying pressure are shown in Fig. 2d. A finite element model of the simplified remote tactile sensing system was developed for the correlation between pressure and magnetic distance using COMSOL Multiphysics 5.2 (hyperelastic module). The upper cap was made of PDMS (thickness, 500 μm; diameter, 9 mm) and the lower cap was made of Ecoflex (thickness, 500 μm; diameter, 7 mm). As external pressure is applied to the top surface of the upper cap, the lower cap is pushed downward. The simulated displacement of the lower cap is shown by the red dotted line. The simulation data indicate that the permanent magnet displacement under external pressure (P) below 400 kPa is limited within 3 mm with the fitting function D = h_*n*_(P) (see supplementary materials for function h_*n*_(P)). Experimental displacement of the permanent magnet was obtained under external pressure of 0~400 kPa (circles); the calculated displacement of the permanent magnet is shown by the green solid line. The experimental and calculation data showed good agreement with the simulation data.

**Figure 3:**
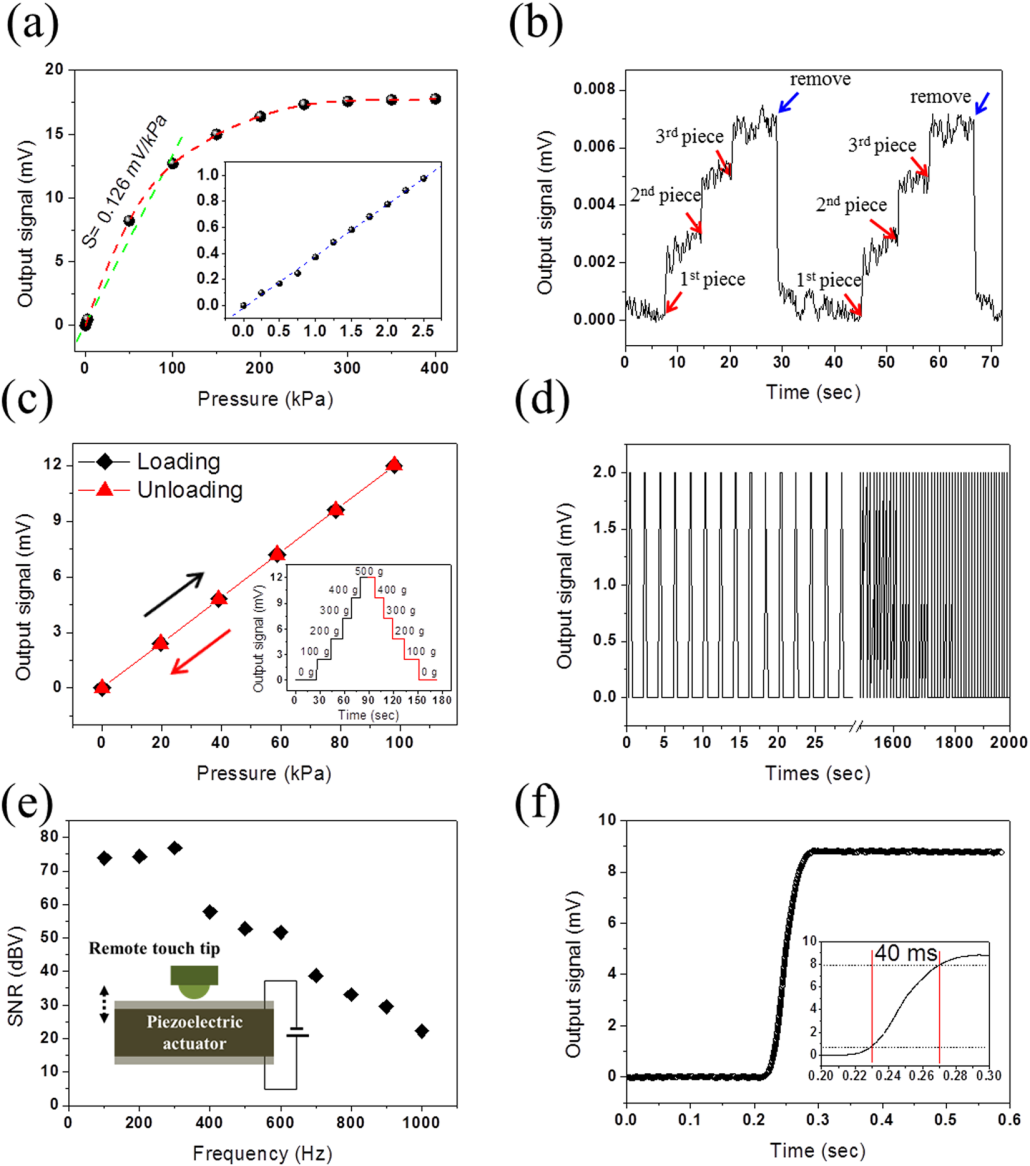
Sensing properties of remote tactile sensing system integrated with magnetic synapse. (**a**) Sensor output signal as a function of applied normal pressure ranging from 0 to 400 kPa; pressure sensitivity S = 0.126 mV/kPa below 100 kPa (inset shows output signal in low pressure range, i.e., 0~2.5 kPa; pressure sensitivity S = 0.4 mV/kPa). (**b**) Detection of minimum distinguishable pressure (6 Pa) using pieces of sandpaper (30 mg each). (**c**) Sensor output during loading and unloading of normal pressure (0~100 kPa) shows no hysteresis. The inset shows the output signal in real-time measurement during loading and unloading (0~500 g). (**d**) Sensing results under repeated normal pressure (40 kPa) for durability (**e**) Signal-to-noise ratio (SNR) versus frequency. The sensor shows high SNR of >70 dBV below 300 Hz and 20 dBV at 1,000 Hz. (**f**) Output signal showing the response time (40 ms). The response time (rise time) is defined as the time interval between 10% and 90% of the state values.

The properties of the developed remote tactile sensing system were characterized using a custom-built system that can apply precise values of pressure to the sensor and measure the output signal in terms of electrical voltage. Figure 3a shows the sensor response under external normal pressure ranging from 0 to 400 kPa. The output signal can be formulated in terms of the external pressure through the correlation function ${\rm{V}}=({{\rm{f}}}_{n}({{\rm{g}}}_{n}({{\rm{h}}}_{n})))={{\rm{F}}}_{n}(P)$ (see supplementary materials for function ${{\rm{F}}}_{n}(P)$) from output signal to magnetic field, magnetic field to magnetic displacement, and magnetic displacement to external pressure. Therefore, the pressure sensitivity ${{\rm{S}}}_{pressure}$ is phenomenologically calculated as the slope of ${{\rm{S}}}_{pressure}$ = dV/dP, and the remote tactile sensing system shows a linear sensitivity of 0.126 mV/kPa up to 100 kPa. Moreover, the remote tactile sensing system can discriminate a very high pressure resolution as it detects the placement and removal of ultralight pieces of sandpaper (30 mg each) on the touch pad (Fig. 3b), which corresponds to 6 Pa. The output signal increased as the pieces of sandpaper were piled up, and it returned to its original value upon their removal; the same results were observed in the repeated experiments (Movie S1).

Very low hysteresis characteristics of the tactile sensor are achieved, whereas it is difficult to do so for tactile sensors made of elastomers. The output signal of the developed tactile sensing system is determined by the position of the magnet relative to the MR sensing element such that there is no direct physical contact with the MR sensor. In addition, the thin membrane where the magnet is fixed shows small hysteresis under external pressure (without distortion). Therefore, the remote tactile sensing system exhibits non-hysteretic behavior under loading and unloading pressure ranging from 0 to 100 kPa, as shown in Fig. 3c. The inset shows that the output signal changes in the time domain during loading and unloading. The durability of the remote tactile sensing system was tested through repeated loading and unloading of 40 kPa at 0.5 Hz, as shown in Fig. 3d. The system showed reliable operation up to 1,000 cycles over 2,000 s (Movie S2).

The frequency response of the developed sensing system was obtained using the test setup shown in Fig. 3e. The piezo-actuator vibrates at the specific frequencies of the applied electrical signal and the remote touch pad responds to the vibration accordingly. The noise level of the developed sensor was −125.55 dBV below 1,000 Hz (Fig. S6). The signal-to-noise ratio (SNR) of the sensor decreased gradually as the frequencies of the applied vibration increased. The SNR was greater than 70 dBV below 300 Hz and 20 dBV at 1,000 Hz. The measured response time (rise time) of the remote tactile sensing system, defined as the time interval between 10% and 90% of the output step height, was 40 ms (Fig. 3f), which is similar value to the response time of human fingertips in the range of 30 to 50 ms^37^. The response time can be further improved in the future by integrating local on-site signal processing circuits with the remote tactile sensing system.

The signal distortion related with the bending curvature of the air tube was examined for robust interconnection in real applications. For the case of bionic arm skin, the signal distortion due to the bending of (articular) joints in the finger and arm originating from the air tube needs to be minimized. Even under extreme conditions, i.e., when the bending curvature of the tube wrapping was 0.04 mm^−1^, the change in the output signal was quite small, i.e., less than 0.005 mV corresponding to 14 Pa (Fig. S7). Therefore, the signal distortion from the bending of the air tube is negligible.

**Figure 4:**
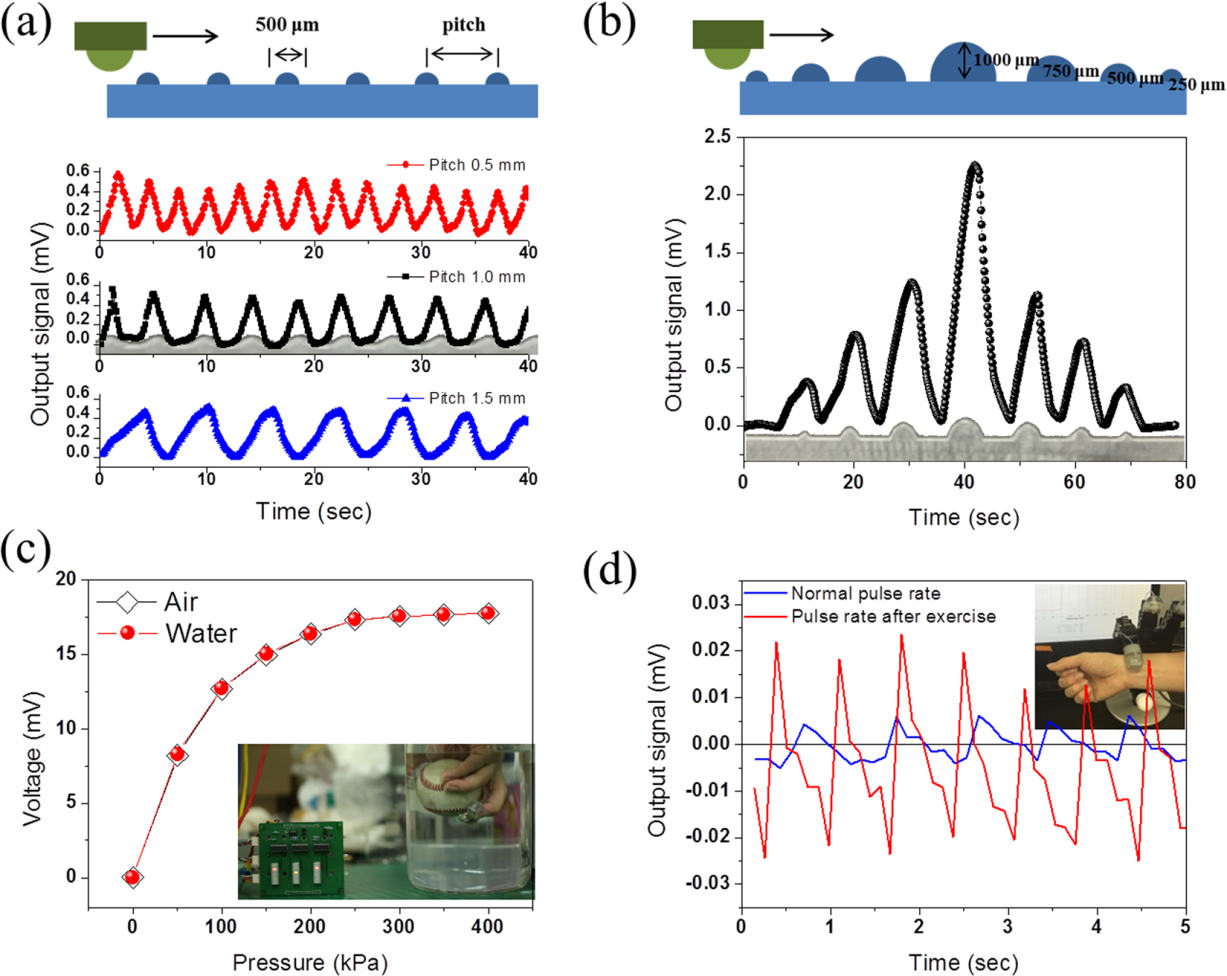
Performance of remote tactile sensing system integrated with magnetic synapse in different applications. (**a**) Different pitches of pattern recognition by the remote tactile sensing system (pattern diameter: 500 μm). (**b**) Different heights of pattern recognition by the remote tactile sensing system (pattern height: 250 μm, 500 μm, 750 μm, and 1,000 μm). (**c**) Sensing signals of the remote tactile sensing system operating in air and water. The inset shows the touch tip attached to fingers holding a baseball in water, and the LED indicates the magnitude of the applied pressure in steps of 50 kPa. (**d**) Measurement results of heart rate and relative blood pressure using the developed remote tactile sensing system under two different conditions: at rest and after exercise. The inset shows the remote tactile sensing system attached to a robot hand (Allegro Hand) to measure the pulse signal automatically in real time.

Real-time texture perception by the tactile sensor was demonstrated using surface patterns with different intervals and heights. Figure 4a shows the result of moving the touch pad on line patterns with different spacings (0.5 mm, 1.0 mm, and 1.5 mm). The cross-sectional diameter of the line pattern was 500 μm. The moving speed of the touch pad was 0.2 mm/s, and the output signal was traced according to the pattern structure. Figure 4b shows the pattern trace of different heights (250, 500, 750, and 1,000 um) with the same pattern spacing.

Figure 4c clearly shows the advantages of the remote tactile sensing system integrated with the magnetic synapse. Conventional tactile sensors have very limited sensing capability in water or extreme environments, as they require extensive packaging of the sensing elements. Our remote tactile sensing system consists of a touch tip that is spatially separated from the sensing magnetic synapse, and the touch tip can be placed and operated in water or extreme environments. The operation of the system in water is shown in Fig. 4c. The sensor signals were nearly identical under external pressure ranging from 0 to 400 kPa in both air and water. The inset shows the submerged touch tips attached to human fingertips holding a baseball in water. According to the magnitude of the pressure applied to the touch tips, the LED shows the pressure level of three remote tactile sensors for grasping (Movie S3). The magnetic synapse also showed high robustness to external vibration noise (Fig. S8). This biomimetic synapse design facilitates robust usage in extreme environments and is not affected significantly by external electrical, magnetic and thermal noise.

In addition, medical diagnosis via pulse measurement from the wrist (real-time monitoring of heart rate and relative blood pressure) was demonstrated using the developed tactile sensing system (Fig. 4d). The tests were carried out by a robot hand under two different conditions: at rest and after physical exercise. The developed remote touch tips were attached to a fingertip of the robot hand (Allegro Hand, Wonik Robotics, Korea) that can bend and unfold its fingers. The remote touch tip measured the heart rate and relative blood pressure as the finger was bent to touch the wrist of a human subject gently, and measurement was ceased when the finger was unfolded, mimicking a doctor’s motion (Movie S4). The touch pressure of the robot finger on the wrist was 10 Pa. The remote tactile sensing system with the robot hand successfully monitored the heart rate and relative blood pressure *in situ* with heart physiology information, as the output signal patterns showed that the heart rate and relative blood pressure increased after exercise.

## Conclusion

In conclusion, a highly sensitive and robust tactile sensing system inspired by the human synaptic system was developed. It offers the advantage of non-direct physical contact between the sensing element and the magnetic synapse system. Physically separating the placement of the remote touch tip and the MR sensing element makes the magnetic synapse system robust against external electromagnetic and thermal noise, thereby facilitating applications in extreme environments and water. The sensitivity and dynamic range of the remote tactile sensing system can be tuned by simply modifying the thickness of the active magnetic and space layers of the MR sensing elements and the initial position of the permanent magnet relative to the MR sensing elements. The developed remote tactile sensing system showed high sensitivity, a wide dynamic range, and excellent durability. Moreover, the magnetic synapse system facilitates hysteresis-free loading and unloading operation. The demonstrated applications of the remote tactile sensor, namely surface texture discrimination, heartbeat measurement, and satisfactory detection quality in water, show that it has considerable potential for use in robotics, medical diagnosis, and prosthetics.

## Methods

### MR sensing element structures

The MR sensing element was fabricated using DC magnetron sputtering and a lift-off process. A photoresist (AZ 5214E) was spin-coated on a Si substrate and soft-baked for 3 min at 100 °C. A chrome mask of a multi-ring pattern was used for the photolithography process. The photoresist of the multi-ring pattern was removed using a developer (AZ 500 MIF) after ultra-violet (UV) exposure, and the remaining photoresist was hard-baked for 2 min at 120 °C. Then, magnetic multilayers (bilayer, spin-valve, and trilayer structure) were deposited by a DC magnetron sputtering system (Fig. S3). The base pressure of the sputtering system was lowered to 3 × 10^−8^ Torr and the sputtering process were carried out at 3 × 10^−3^ Torr. During the deposition, a uniform in-plane magnetic field of 250 Oe was applied to induce an exchange bias field at the interface between ferromagnetic (NiFe) and antiferromagnetic (IrMn).

### Fabrication of the remote tactile sensing system with magnetic synapse

The remote touch tip has an air chamber capped by a PDMS (Dow Corning Corp., Sylgard 184, Auburn, MI, USA) membrane having a hemispherical tip at the center. The air chamber was fabricated by a 3D printer (Objet 30 Pro, STRATASYS Ltd., Eden Prairie, MN, USA). The PDMS base and cross-linker were mixed (10:1) and stirred for 5 min; the mixture was degassed under vacuum for 20 min and then casted on the VeroClear (RGD 810, STRATASYS Ltd., Eden Prairie, MN, USA) master. The master was modified, i.e., Ag was deposited by a sputtering system and 1H, 1H, 2H,2H-perfluoro-octyltriethoxysilane was vapor-deposited (Sigma-Aldrich, Inc., St. Louis, USA). The thickness and diameter of the PDMS membrane were 500 μm and 9 mm, respectively, and the hemispherical tip thickness and diameter were 7 mm and 8 mm, respectively. The magnetic synapse consists of an Ecoflex 0030 (Smooth-On Inc., Macungie, PA, USA) membrane having a thickness of 500 μm and a diameter of 7 mm with a permanent magnet inserted over a magnetoresistive (MR) sensing element. PTFE air tubes having an outer diameter of 2 mm and inner diameter of 1 mm were used. Finite element simulation (ANSYS, Maxwell 16.1) was carried out to analyze the initial position of a cylindrical permanent magnet (ϕ3 mm × 2 mm) with the magnetic synapse. The permanent magnet was embedded in the membrane and moved toward the MR sensing element as air pressure was applied to the membrane. To optimize the remote tactile sensing system, the initial vertical distance of the permanent magnet relative to the MR sensing element should be less than 3 mm.

### Air tube bending

Three types of polytetrafluoroethylene (PTFE) tubes with an outer diameter of 2 mm and inner diameters 1.8 mm, 1.4 mm, and 1.0 mm were used to investigate the effect of air tube bending. With a modulus of 0.5 GPa for the 0.5-mm-thick tube, a curvature of 0.02 mm^−1^ of the tube, corresponding to wrapping around a glass bottle having a diameter of 42 mm, caused a change of less than 0.005 mV in the output signal, corresponding to 14 Pa. As the wall thickness of the air tube decreases, the output voltage becomes more susceptible to tube bending in the remote sensing element. Even under an extreme bending curvature of 0.04 mm^−1^ for the tube wrapping with a thickness of 0.1 mm, the change in the output signal was quite small, i.e., <0.057 mV corresponding to 150 Pa (Fig. S7).

### Measurement of sensing performance of the remote tactile sensing system integrated with magnetic synapse

The developed remote tactile sensing system was characterized with a custom-built tactile characterization system, which can apply precise values of pressure to the sensor and measure the output signal in terms of electrical voltage (NI DAQ 9234, National Instruments Corp., Austin, TX, USA). The system can apply pressure in the range of 0 to 2,500 gf in steps of 25 gf. The MR sensing element was characterized under an external magnetic field in the range of −300 to +300 Oe. The external magnetic field was generated by a Helmholtz coil (custom-built) to change the magnetization direction of the free layer as well as the magnet field strength. The output signal of the MR sensing element was characterized using a sourcemeter (Keithley 2450, Tektronix, Inc., Solon, Ohio, USA) with a source current of 1 mA. The output signal variation of the remote sensing system under different temperature conditions was checked in Fig. S9.

### Participants

Measurement of heart rate and relative blood pressure using the developed remote tactile sensing system was performed with one of author. The Ethics committee of Daegu Gyeongbuk Institute of Science and Technology (DGIST) approve the study, and all methods were performed in accordance with ethical guidelines and regulations as offered by the DGIST.

## Electronic supplementary material

Supplementary Information

Video 1

Video 2

Video 3

Video 4
