# Supplementary material for: Remote tactile sensing system integrated with magnetic synapse
Source: Sci Rep. 2017 Dec 5;7:16963. doi: 10.1038/s41598-017-17277-2 (PMC5717174; doi:10.1038/s41598-017-17277-2)
Supplement: Supplementary file 1 — Supplementary Information [file 41598_2017_17277_MOESM1_ESM.pdf]

Supplementary Information

Remote tactile sensing system integrated with magnetic synapse

Sunjong Oh[1], Youngdo Jung[1], Seonggi Kim[1], SungJoon Kim[2], Xinghao Hu[2], Hyuneui Lim[1,*], CheolGi Kim[2,*]

[1] Department of Nature-Inspired Nanoconvergence Systems, Korea Institute of Machinery and Materials, Daejeon 356248, Republic of Korea

[2] Department of Emerging Materials Science & Center for Bio-Convergence Spin System, DGIST, Daegu 42988, Republic of Korea

Correspondence and requests for materials should be addressed to Hyuneui Lim (email: helim@kimm.re.kr) or CheolGi Kim (email: cgkim@digst.ac.kr).

Table S1. A summarization of specifications of minimum detectable weight and dynamic pressure range of several pressure sensors.

References	Minimum detectable weight	Pressure range
1)	20 mg(fly)	3 Pa ~ 20 kPa
2)	30 mg(clip)	1.5 Pa ~ 1 kPa
3)	20 mg(gas pressure)	0.6 Pa ~ 49.5 kPa
4)	400 mg(metal bar)	20 Pa ~ 1 kPa
5)	1 mg(ant)	0.2 Pa ~ 10 kPa
**This work**	**30 mg(paper)**	**6 Pa ~ 400 kPa**

References

1) Mannsfeld, S. C. *et al.* Highly sensitive flexible pressure sensors with microstructured rubber dielectric layers. *Nat Mater* **9**, 859–864 (2010).

2) Zhu, B. *et al.* Microstructured graphene arrays for highly sensitive flexible tactile sensors. *Small* **10**, 3625-3631 (2014).

3) Park, J. *et al.* Fingertip skin-inspired microstructured ferroelectric skins discriminate static/dynamic pressure and temperature stimuli. *Sci. Adv.* **1**, e1500661 (2015).

4) Lee, S. *et al.* A transparent bending-insensitive pressure sensor. *Nat Nanotech.* **11**, 472-479 (2016).

5) Choi, Y. *et al.* Ultra-sensitive pressure sensor based on guided straight mechanical cracks. **7**, 40116 (2017).

The remote tactile sensing system consists of a remote touch tip that generates air pressure under external stimulation, an air tube that delivers the air pressure, and a magnetic synapse that transduces the air pressure into electrical signals. The remote touch tip has an air chamber covered with a PDMS membrane having a hemispherical tip at the center. The thickness and diameter of the PDMS membrane are 500 μm and 9 mm, respectively. The height of the hemispherical tip is 7 mm. Under external stimulation of the remote touch tip, the air chamber is compressed by deformation of the PDMS membrane. The compressed air is transferred through the air tube to the magnetic synapse. The air tube is made of PTFE with an outer diameter of 2 mm and an inner diameter of 1 mm. The magnetic synapse consists of an Ecoflex 0030 membrane having a thickness of 500 μm and a diameter of 7 mm with a permanent magnet at the center and a multi-ring magnetoresistive (MR) sensing element beneath the membrane.

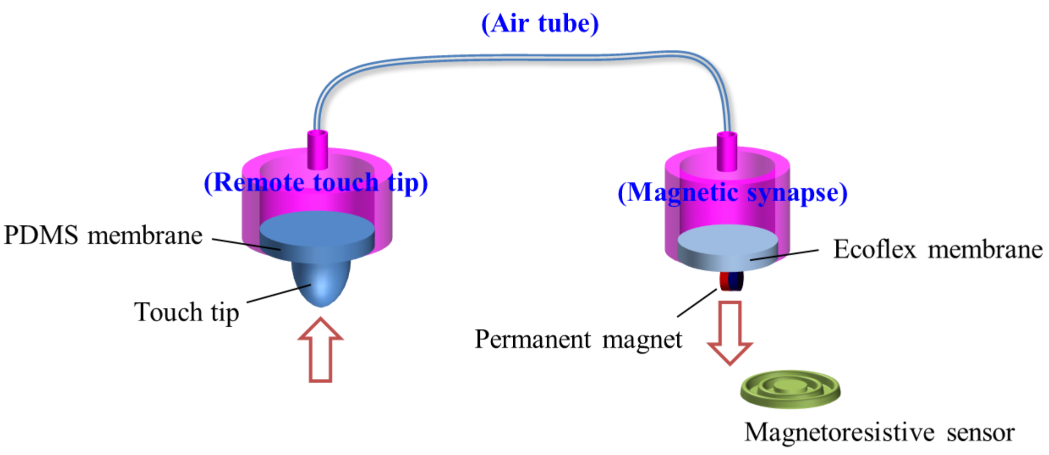

Fig. S1. Schematic diagram of remote tactile sensing system with magnetic synapse and principle of operation of the remote tactile sensing system.

The sensitivity of the remote tactile sensing system can be modified by the Young's modulus of the elastomer membrane in the remote touch tip. The remote tactile sensing system with an Ecoflex (Young`s modulus: 44 kPa) membrane exhibited sensitivity four times higher than that with a PDMS (Young`s modulus: 833 kPa) membrane.

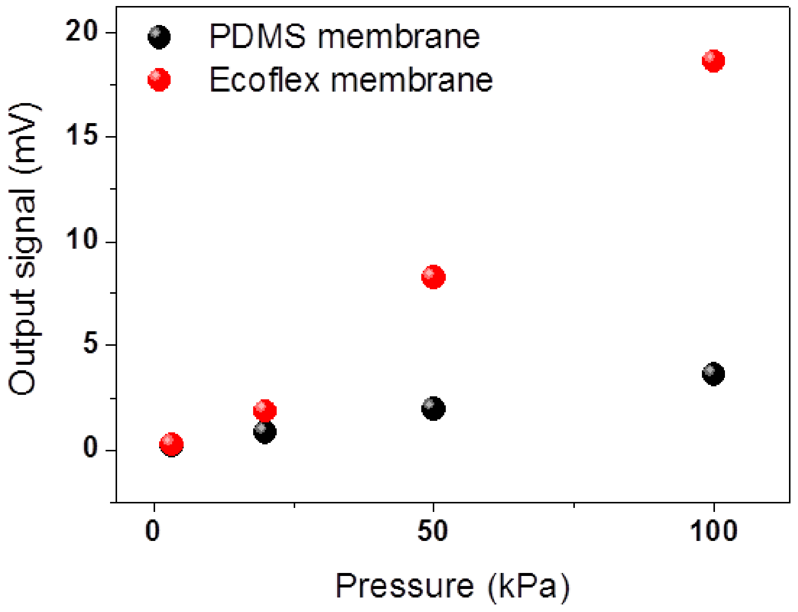

Fig. S2. Sensor output signal versus applied normal pressure ranging from 0 to 100 kPa with different membranes.

The output signal of the MR sensing element is determined by the external magnetic field strength and is described as

$$V = {}^{I}/_{t}\,\Delta\rho \sin\theta \cos\theta \tag{5}$$

$$\theta = f(H) \tag{6}$$

where I and t are the source current and thickness of the ferromagnetic layer, and $\Delta\rho = \rho_{\parallel} - \rho_{\perp}$, where $\rho_{\parallel}$ and $\rho_{\perp}$ are the electrical resistivity when the magnetization direction of the ferromagnetic layer without the external magnetic field and the source current direction are parallel and perpendicular to each other, respectively. The angle between the current and the magnetization direction is a function of the external magnetic field strength (H) at the MR sensing element.

Three different MR sensing elements were fabricated using DC magnetron sputtering: a bilayer thin film Ta(3 nm)/NiFe(10 nm)/ IrMn(10 nm)/Ta(3 nm), a spin-valve thin film Ta(3 nm)/NiFe(10 nm)/ Cu(1.2 nm)/NiFe(2 nm)/IrMn(10 nm)/Ta(3 nm), and a trilayer thin film (3 nm)/NiFe(10 nm)/Cu(0.12 nm)/IrMn(10 nm)/Ta(3 nm) with MR sensing elements of multi-ring structures (diameter, 300 μm). The field sensitivities of the MR sensing elements based on the bilayer, spin-valve, and trilayer thin films are 0.39, 1.15, and 2.79 mV/Oe, respectively.

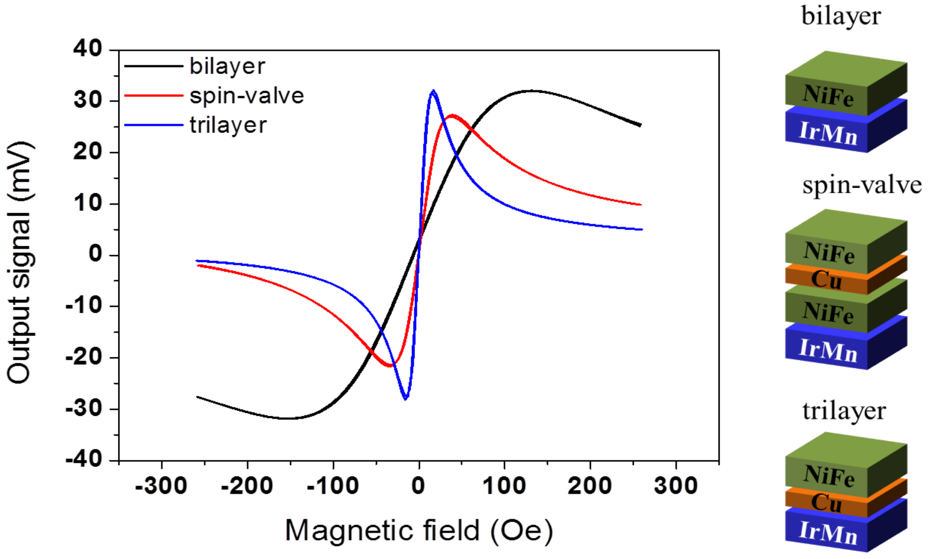

Fig. S3. Three types of MR sensing elements consisting of a bilayer Ta(3 nm)/NiFe(10 nm)/ IrMn(10 nm)/Ta(3 nm), a spin-valve Ta(3 nm)/NiFe(10 nm)/ Cu(1.2 nm)/NiFe(2 nm)/IrMn(10 nm)/Ta(3 nm), and a trilayer structure (3 nm)/NiFe(10 nm)/Cu(0.12 nm)/IrMn(10 nm)/Ta(3 nm). The magnetic field sensitivity was 0.39, 1.15, and 2.79 mV/Oe in the range of ±300 Oe.

The remote sensing system with the MR sensing element based on a trilayer structure has the highest sensitivity of 0.224 mV/kPa, and the measurable pressure range is 0 to 100 kPa. The output signal of the MR sensing element decreases in the pressure range above the measureable range of 100 kPa. The magnetic field sensitivities of the MR sensing elements with spin-valve and bilayer structures are 0.126 mV/kPa and 0.065 mV/kPa lower than that with the trilayer structure, respectively, but the measurable range is extended to 400 kPa.

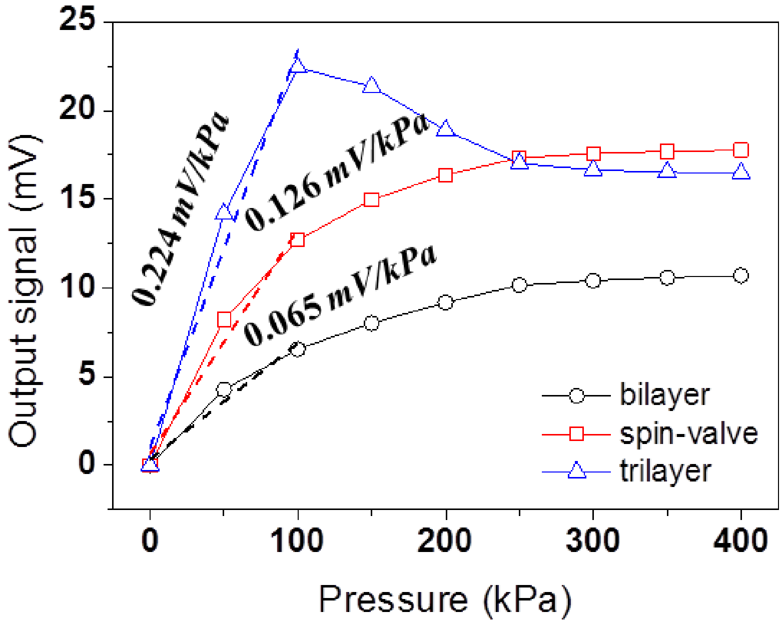

Fig. S4. Sensor output signal according to MR sensing element structure (normal pressure ranging from 0 to 400 kPa). The pressure sensitivity of the bilayer, spin-valve, and trilayer structure is 0.065 mV/kPa, 0.126 mV/kPa, and 0.224 mV/kPa below 100 kPa.

From the fitting functions of correlating output signal (V) to magnetic field (H), magnetic field (H) to magnet displacement (D), and magnetic displacement (D) to external pressure (P), the output signal can be formulated in terms of external pressure as followings;

$$\mathrm{V} = \mathrm{f}_n(H) \cong 0.001\,H \qquad (1)$$

$$\mathrm{H} = \mathrm{g}_n(D) \cong a_0 + a_1 D^1 + a_2 D^2 \qquad (2)$$

$$\mathrm{D} = \mathrm{h}_n(P) \cong b_0 + b_1 P^1 + b_2 P^2 \qquad (3)$$

$$\mathrm{V} = (\mathrm{f}_n(\mathrm{g}_n(\mathrm{h}_n))) = \mathrm{F}_n(P) \approx c_0 + c_1 P^1 + c_2 P^2 + c_3 P^3 + c_4 P^4 \qquad (4)$$

Fig. S5 shows a simulated magnetic field produced by a cylindrical permanent magnet (3 mm X 2 mm). The vector color visualizes the strength of the magnetic field and the exact directions of the magnetic field on the MR sensor. The field directions are changed based on the relative position of the MR sensor to the permanent magnet. Each dot in the magnetic field chart shows the position of the MR sensor and the arrow show the magnetic field direction exerted on the MR sensor.

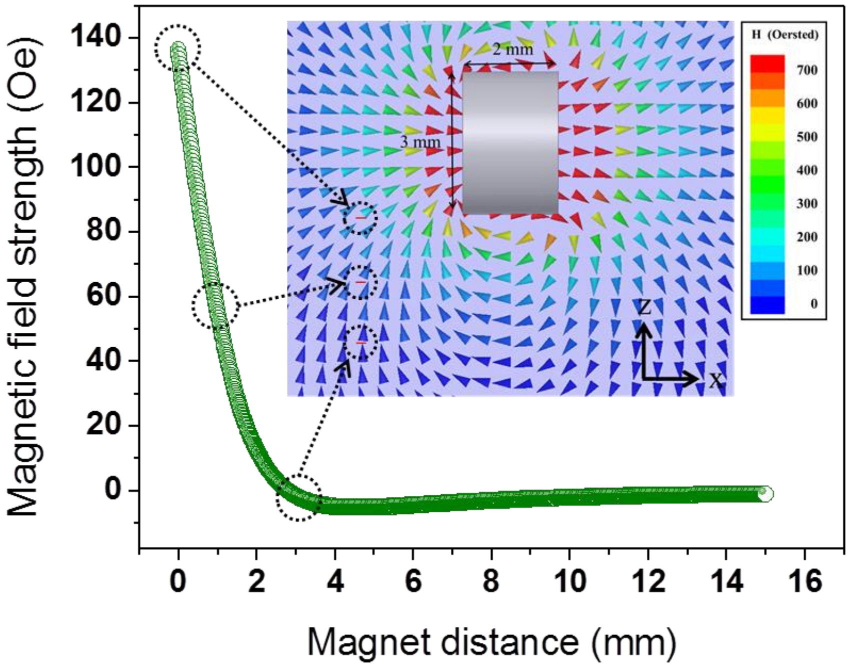

Fig. S5. Finite element simulation (ANSYS, Maxwell 16.1) showing the correlation between the vertical position of the permanent magnet and the magnetic field strength present at the MR sensing element.

The frequency response of the developed sensing system was tested with the test setup shown in Fig. S6. The remote touch tip was placed on a piezoelectric actuator vibrating in the vertical direction at specific frequencies of the applied electrical signal. The vertical vibration was transferred to the remote sensing system by the magnetic synapse through the remote touch tip. The measured noise level of the developed sensor was around -125.55 dBV. The output voltage of the sensor was 70 dBV higher than the noise floor at 300 Hz and 20 dBV at 1,000 Hz.

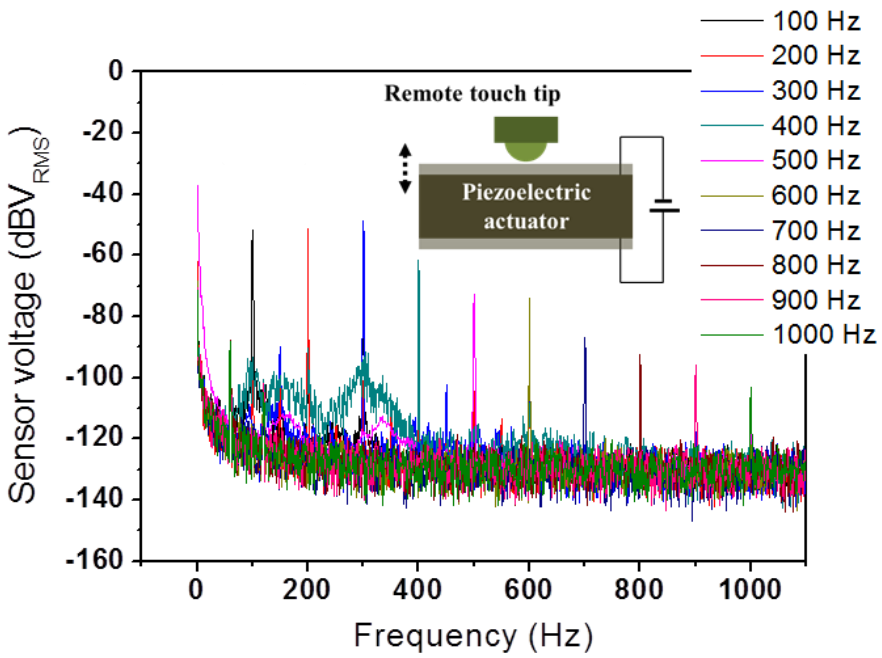

Fig. S6. Frequency characteristics of the remote sensing system with magnetic synapse up to 1,000 Hz.

The signal distortion due to the bending of (articular) joints in the finger and arm needs to be minimized. The signal distortion related with the bending curvature of the air tube was examined with different wall thicknesses. PTFE tubes (ZEUS, Inc., Orangeburg, SC, USA) with an outer diameter of 2 mm and inner diameters 1.8 mm, 1.4 mm, and 1.0 mm were used. With a modulus of 0.5 GPa for the 0.5-mm-thick tube, a curvature of 0.02 mm^{-1} of the tube, corresponding to wrapping around a glass bottle having a diameter of 42 mm, caused a change of less than 0.005 mV in the output signal, corresponding to 14 Pa. As the wall thickness of the air tube decreases, the output voltage becomes more susceptible to tube bending in the remote sensing element. Even under an extreme bending curvature of 0.04 mm^{-1} for the tube wrapping with a thickness of 0.1 mm, the change in the output signal was quite small, i.e., <0.057 mV corresponding to 150 Pa (Fig. S7).

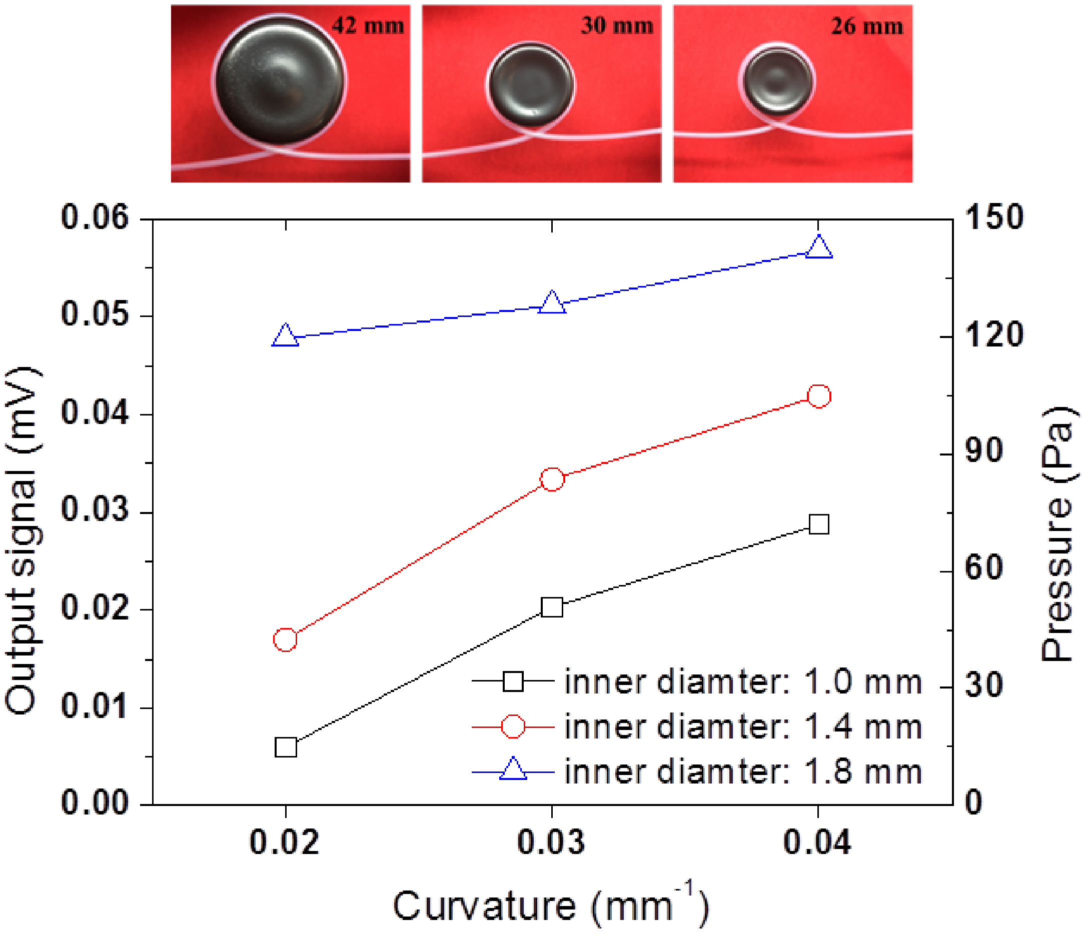

Fig. S7. Signal variation of remote sensing system with magnetic synapse according to inner diameter and wrapping curvature of air tube.

The sensor part (magnetic synapse) was placed on the vibrating box actuated by a vibratory speaker to test the robustness of the system to external vibration noise. The noise signal was measured, while the sensor part was vibrating up-and-down vigorously. The measured noise signal was less than 30 uV, which is only 0.17% of the maximum signal change by pressure (17.8 mV).

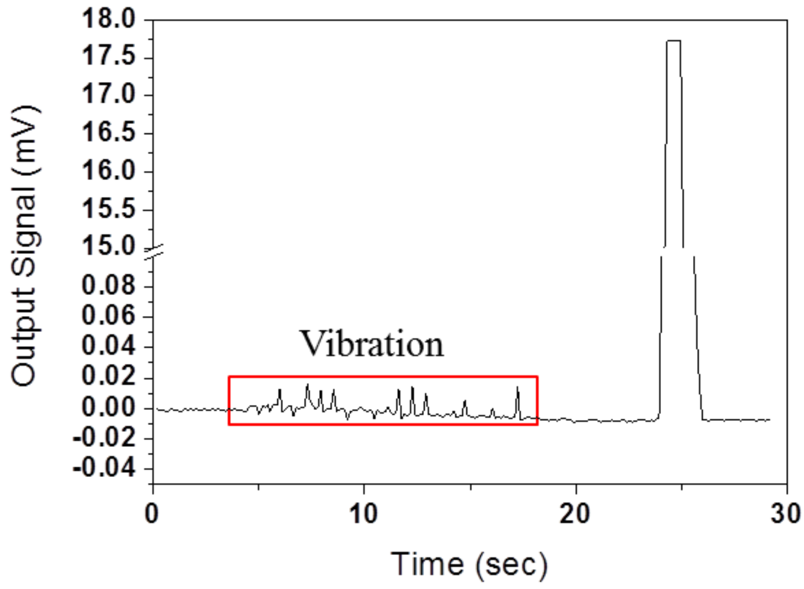

Fig. S8. Signal changes due to external vibration of the sensor part (magnetic synapse).

Fig. S9 shows the output signal variation of the remote sensing system under different temperature conditions. The remote touch tip was attached to a customized tactile characterization system equipped with the Peltier device. As the level of customized tactile characterization system was lowered, the remote touch tip was pressed against the Peltier device. Therefore, the temperature of remote touch tip was changed by connecting with Peltier, which can change its surface temperature. The change in the output signal between -10°C and 80°C without applied pressure of the remote touch tip was 0.80 mV, corresponding to a pressure of 2 kPa. The air in the chamber of the remote touch tip shrinks or expands owing to temperature changes, resulting in drift of the output signal. However, the changes are much smaller compared to the cases in which the MR sensing elements are exposed to temperature changes directly.

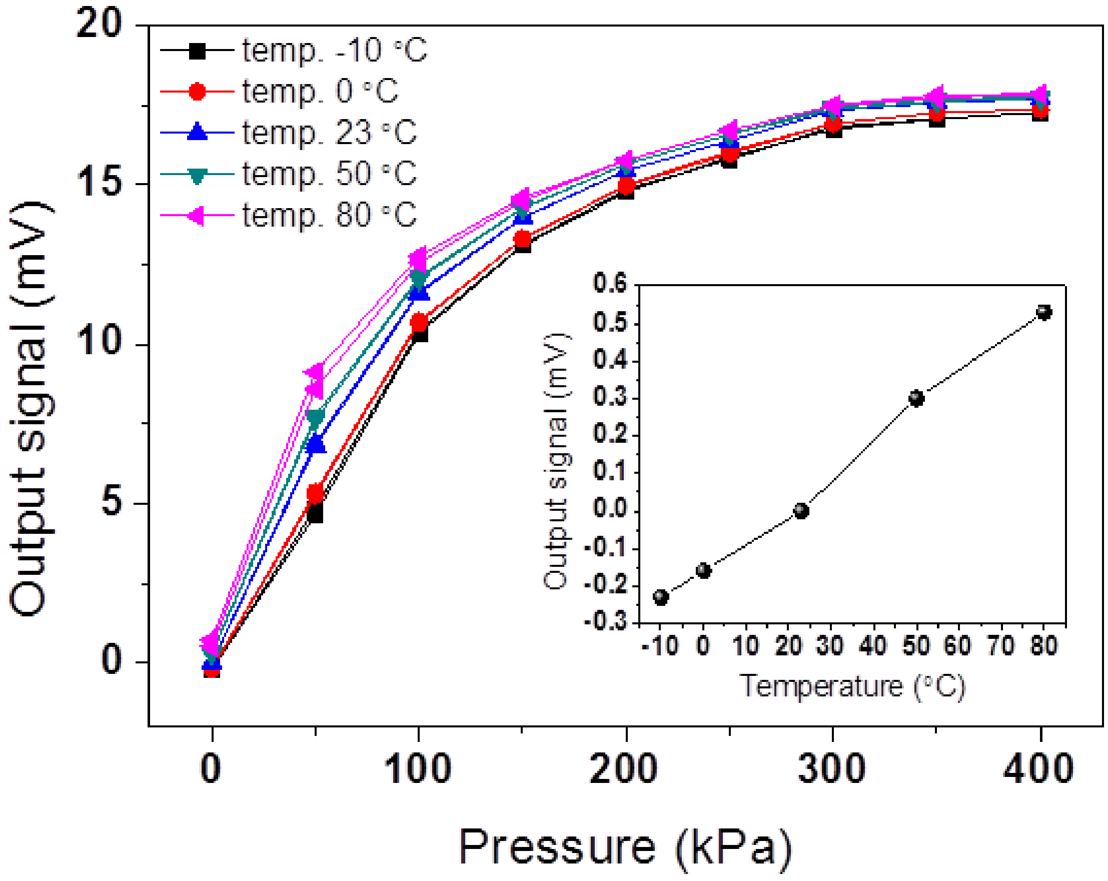

Fig. S9. Sensor output signal under temperatures ranging from -10°C to 80°C and normal pressure ranging from 0 to 400 kPa. The signal variation without pressure was 0.80 mV corresponding to a pressure of 2 kPa.
